# Canstatin inhibits hypoxia-induced apoptosis through activation of integrin/focal adhesion kinase/Akt signaling pathway in H9c2 cardiomyoblasts

**DOI:** 10.1371/journal.pone.0173051

**Published:** 2017-02-24

**Authors:** Hiroki Kanazawa, Keisuke Imoto, Muneyoshi Okada, Hideyuki Yamawaki

**Affiliations:** Laboratory of Veterinary Pharmacology, School of Veterinary Medicine, Kitasato University, Towada, Aomori, Japan; University of Cincinnati College of Medicine, UNITED STATES

## Abstract

A hypoxic stress which causes apoptosis of cardiomyocytes is the main problem in the ischemic heart disease. Canstatin, a non-collagenous fragment of type IV collagen α2 chain, is an endogenous anti-angiogenic factor. We have previously reported that canstatin has a cytoprotective effect on cardiomyoblasts. In the present study, we examined the effects of canstatin on hypoxia-induced apoptosis in H9c2 cardiomyoblasts. Cell counting assay was performed to determine a cell viability. Western blotting was performed to detect expression of cleaved casepase-3 and phosphorylation of focal adhesion kinase (FAK) and Akt. Immunocytochemical staining was performed to observe a distribution of α_v_ integrin. Hypoxia (1% O_2_, 48 h) significantly decreased cell viability and increased cleaved caspase-3 expression. Canstatin (10–250 ng/ml) significantly inhibited these changes in a concentration-dependent manner. Cilengitide (1 μM), an α_v_β_3_ and α_v_β_5_ integrin inhibitor, significantly prevented the protective effects of canstatin on cell viability. Canstatin significantly increased phosphorylation of FAK and Akt under hypoxic condition, which were inhibited by cilengitide. LY294002, an inhibitor of phosphatidylinositol-3 kinase/Akt pathway, suppressed the canstatin-induced Akt phosphorylation and reversed the protective effects of canstatin. It was observed that hypoxia caused a localization of α_v_ integrin to focal adhesion. In summary, we for the first time clarified that canstatin inhibits hypoxia-induced apoptosis via FAK and Akt pathways through activating integrins in H9c2 cardiomyoblasts.

## Introduction

Ischemic heart disease such as myocardial infarction is one of the leading causes for death throughout the world [1–3]. A hypoxic stress is the main problem in the ischemic heart disease, which induces apoptosis through the activation of caspase-cascade by a release of cytochrome c from mitochondria to cytoplasm [4,5]. Cell death of matured myocardial cells directly causes a fatal cardiac dysfunction [6–8]. Therefore, the control of the hypoxia-induced apoptosis in cardiomyocytes is thought to be an important therapeutic strategy in the treatment of ischemic heart disease.

Type IV collagen, a major component of the basement membrane, consists of a triple helical structure [9,10]. Canstatin, a non-collagenous fragment, is cleaved from type IV collagen α2 chain, an essential component of basement membrane surrounding cardiomyocytes [11–18]. It is presumed that canstatin exerts anti-angiogenic and anti-tumor effects through binding its receptor, α_v_β_3_ and α_v_β_5_ integrins, in endothelial and tumor cells [11–16]. α_v_β_3_ and α_v_β_5_ integrins are colocalized with human coxsackie-adenovirus receptor on the cardiomyocyte sarcolemma in a dilated cardiomyopathy patient [19]. In situ hybridization revealed that intense expression of α_v_ integrin mRNA was seen in rat cardiomyocytes [20]. In primary adult rat ventricular cardiomyocytes, α_v_ and β_1_, β_3_ or β_5_ integrins are required in periostin-induced cardiomyocyte cell-cycle reentry [21]. It has been reported that cell surface expression of α_v_β_3_ and α_v_β_5_ integrins is increased by hypoxia stimulation in tumor cells [22,23]. β_3_ integrin prevents oxidative stress-induced apoptosis in HL-1 mouse cardiomyocyte cell line [24]. It has also been reported that α_v_β_3_ and α_v_β_5_ integrins activated the survival signaling pathway through the activation of Akt [25–27] which was responsible for the protection against ischemia-reperfusion injury in mouse cardiomyocytes [28]. In addition, we previously reported that canstatin inhibited isoproterenol-induced apoptosis of differentiated H9c2 cardiomyoblasts [29]. Thus, it is suggested that canstatin might be an endogenous cardioprotective factor in cardiomyocytes. In this study, we tested the hypothesis that canstatin affects hypoxia-induced apoptosis of cardiomyoblasts through the integrins/Akt pathways.

## Materials and methods

### Reagents and antibodies

Reagent sources were as follows: recombinant mouse collagen alpha-2 (IV) chain, partial (canstatin) (Cusabio Life science, Hubei, China), cilengitide (Adooq Bioscience, Irvine, CA, USA), LY294002 (Wako, Osaka, Japan). Antibodies sources were as follows: phospho-focal adhesion kinase (FAK) (Ser397), total-FAK, α_v_ integrin, vinculin (Santa Cruz Biotechnology, Santa Cruz, CA, USA); phospho-Akt (Ser473), cleaved caspase-3, total-Akt (Cell Signaling Technology, Beverly, MA, USA), total-actin (Sigma-Aldrich, St. Louis, MO, USA)

### Hypoxic conditioning

H9c2 cardiomyoblasts obtained from American Type Culture Collection (CRL-1446; Manassas, VA, USA) were cultured in Dulbecco Modified Eagle's Medium (DMEM; Wako, Osaka, Japan) supplemented with 10% fetal bovine serum (FBS; Invitrogen, Carlsbad, CA, USA or HyClone/GE Healthcare, Little Chalfont, UK) and a mixture of 1% penicillin-streptomycin-amphotericin B (Nacalai Tesque, Kyoto, Japan). H9c2 cells at confluence were starved with serum-free DMEM and incubated in a normoxic condition (20% O_2_, 5% CO_2_, 37°C) or a hypoxic condition (1% O_2_, 5% CO_2_, 37°C) in the presence of varying drugs.

### Phase contrast microscopy

H9c2 cardiomyoblasts were grown to confluent and stimulated with canstatin (10–250 ng/ml) or vehicle of canstatin (20 mM Tris, 500 mM L-arginine, 50% glycerol, pH 8.0 for control group) for 48 h. Cell morphology was observed with a phase contrast microscope (CKX-31, OLYMPUS, Tokyo, Japan).

### Cell counting assay

Living cell number was counted by a cell counting kit 8 (CC8; Dojindo, Kumamoto, Japan) as described previously [30]. After the stimulation with hypoxia (48 h) in the presence or absence of canstatin (10–250 ng/ml), the cells were washed with Tris-buffered saline (TBS, pH 7.4) and treated with CC8 solution (50 μl/1.0 ml medium) for 1 h at 37°C. The absorbance of the media at 485 nm was measured using a standard microplate reader (Tristar, Berthold Technology, Bad Wildbad, Germany).

### Western blotting

Western blotting was performed as described previously [30]. After the stimulation of the cells with hypoxia (24–48 h) in the presence or absence of canstatin (10–250 ng/ml), cilengitide (1 μM) and/or LY294002 (10 μM), total cell lysates were harvested by homogenizing the cells with Triton X-100-based lysis buffer (Cell Signaling Technology) with a protease inhibitor cocktail (Nacalai Tesque). Protein concentration was determined using a bicinchoninic acid method (Pierce, Rockfold, IL, USA). Equal amounts of proteins (10 μg) were separated by sodium dodecyl sulfate-polyacrylamide gel electrophoresis (SDS-PAGE) (7.5–14%) and transferred to nitrocellulose membrane (Pall Corporation, Ann Arbor, MI, USA) or polyvinylidene fluoride membrane (ATTO, Tokyo Japan). After being blocked with 0.5% skim milk (for total proteins) or 3% bovine serum albumin (for phosphorylated proteins), the membranes were incubated with primary antibody overnight at 4°C. After incubation with secondary antibody, the chemiluminescence signal was detected by EZ-ECL Western blotting detection reagents (Biological Industries, Kibbutz Beit, Haemek, Israel) using the ATTO light capture system (AE- 6972; ATTO Co., Tokyo, Japan).

### Immunocytochemical staining

Immunocytochemical staining was performed as described previously [31]. Twenty four hours after incubation in a normoxic condition (20% O_2_, 5% CO_2_, 37°C) or a hypoxic condition (1% O_2_, 5% CO_2_, 37°C), the H9c2 cardiomyoblasts were fixed with 4% paraformaldehyde at 4°C for 10 min. Then the cells were permeabilized with 0.2% Triton X-100 (Sigma-Aldrich) at room temperature for 1 min. The permeabilized cells were blocked with 5% normal goat serum for 1 h and incubated with anti-α_v_ integrin and anti-vinculin antibody overnight at 4°C. The cells were incubated with Alexa 488 dye conjugated goat anti-rabbit IgG at room temperature for 1 h. The images were blindly captured using TrueChromeII Plus (BioTool, Gunma, Japan).

### Statistical analysis

Data were shown as mean ± standard error of the mean (S.E.M). Statistical evaluations were performed by one-way analysis of variance (ANOVA) followed by Bonferroni's post hoc test. A value of p<0.05 was considered as statistically significant.

## Results

### Canstatin inhibits hypoxia-induced apoptosis of H9c2 cardiomyoblasts

**Fig 1:**
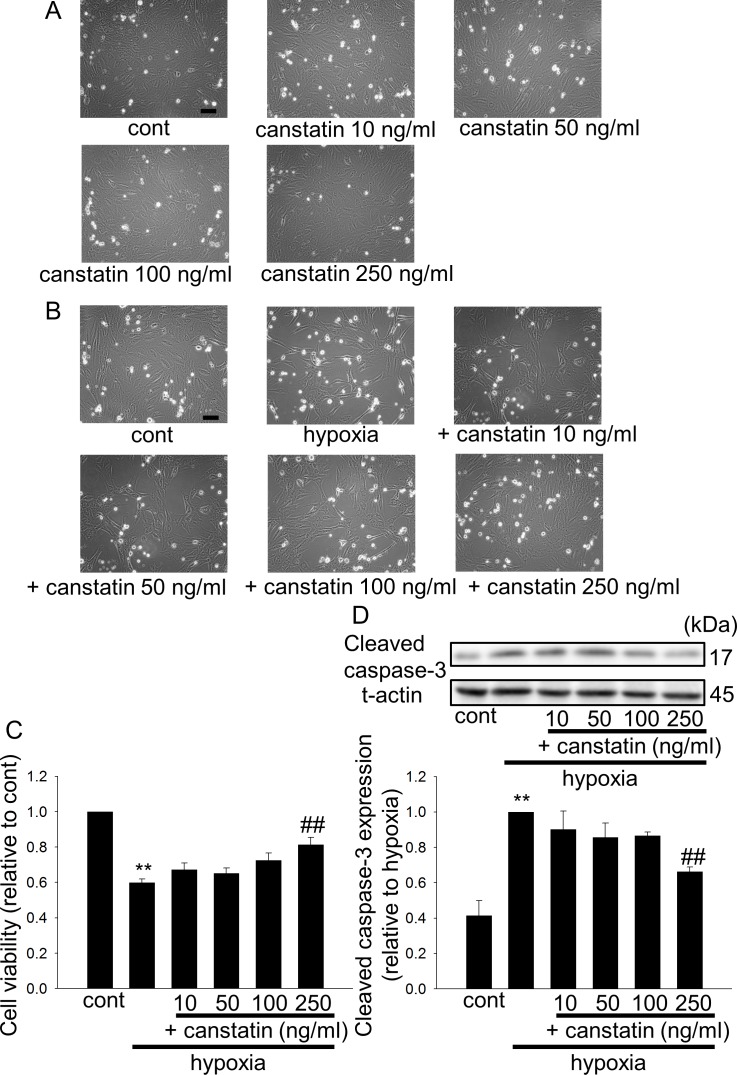
Canstatin inhibits hypoxia-induced apoptosis of H9c2 cardiomyoblasts. (A) H9c2 cardiomyoblasts were treated with canstatin (10–250 ng/ml) for 48 h under normoxia (20% O_2_, 5% CO_2_, 37°C). Representative phase-contrast microscopic images of H9c2 cells after vehicle (cont) or canstatin-alone treatment (10–250 ng/ml, 48 h) were shown (n = 3). Scale bar represents 100 μm. (B-D) H9c2 cardiomyoblasts were stimulated with hypoxia for 48 h in the presence or absence of canstatin (10–250 ng/ml). (B) Representative phase-contrast microscopic images of H9c2 cells after treatment with vehicle (cont), hypoxia-alone, or hypoxia + canstatin (250 ng/ml) were shown (n = 7). Scale bar represents 100 μm. (C) After the stimulation with hypoxia, living cell number was counted by a colorimetric method using cell counting kit-8. The normalized cell number relative to cont was shown as mean ± standard error of the mean (S.E.M.). (n = 5). **: p<0.01 vs. cont, ##: p<0.01 vs. hypoxia. (D) After the stimulation with hypoxia, total cell lysates of H9c2 cardiomyoblasts were harvested. Expression of cleaved caspase-3 and total actin (t-actin) was determined by Western blotting. Representative blots of cleaved casepase-3 and t-actin were shown (upper). Levels of cleaved caspase-3 were corrected by t-actin, and the normalized expression relative to hypoxia-alone treatment was shown as mean ± S.E.M. (n = 5). **: p<0.01 vs. cont, ##: p<0.01 vs. hypoxia (lower).

We first confirmed that canstatin-alone treatment (10–250 ng/ml, 48 h) had no effect on the morphology of H9c2 cardiomyoblasts under normoxia (Fig 1A, n = 3). We also confirmed that a higher concentration of canstatin (1 μg/ml) under normoxia had no effect on cell viability (data not shown, n = 6). We next examined the effects of canstatin on hypoxia-induced morphological changes in H9c2 cardiomyoblasts. Morphological changes of cell death, such as detachment of cells, were observed in hypoxia (48 h)-stimulated cells, which were suppressed by canstatin (10–250 ng/ml) in a concentration-dependent manner (n = 7, Fig 1B). We also found that hypoxia (48 h) significantly decreased cell viability (p<0.01 vs. cont), which was significantly suppressed by canstatin in a concentration-dependent manner (10–250 ng/ml) (n = 5, p<0.01 vs. hypoxia, Fig 1C). Canstatin also inhibited the hypoxia (48 h)-induced increases of cleaved caspase-3 expression (p<0.01 vs. cont) in a concentration-dependent manner (10–250 ng/ml) (n = 5, p<0.01 vs. hypoxia, Fig 1D).

### Canstatin suppresses hypoxia-induced apoptosis in H9c2 cardiomyoblasts through activating integrins

**Fig 2:**
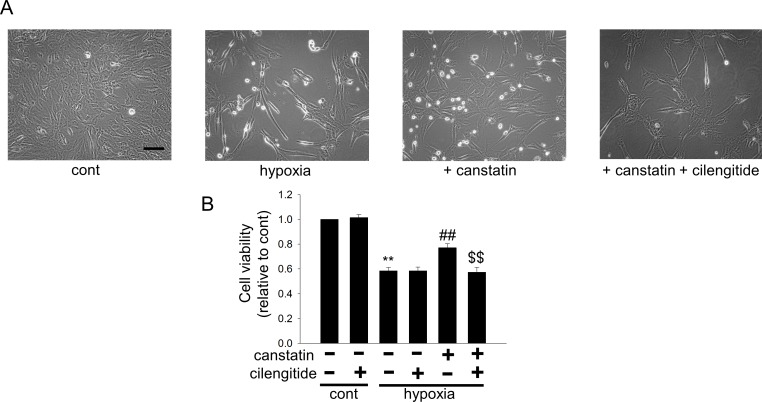
Canstatin suppresses hypoxia-induced apoptosis in H9c2 cardiomyoblasts through the activation of α_v_β_3_ and/or α_v_β_5_ integrins. H9c2 cardiomyoblasts were stimulated with hypoxia for 48 h in the presence or absence of canstatin (10–250 ng/ml) and cilengitide (1 μM), an α_v_β_3_ and/or α_v_β_5_ integrin inhibitor. (A) Representative phase-contrast microscopic images of H9c2 cells after treatment with vehicle (cont), hypoxia-alone, hypoxia + canstatin (250 ng/ml), or hypoxia + canstatin (250 ng/ml) + cilengitide (1 μM) were shown (n = 5). Scale bar represents 100 μm. (B) After the stimulation with hypoxia for 48 h in the presence or absence of canstatin and cilengitide, living cell number was counted by a colorimetric method using cell counting kit-8. The normalized cell number relative to cont was shown as mean ±S.E.M (n = 5). **: p<0.01 vs. cont, ##: p<0.01 vs. hypoxia, $$: p<0.01 vs. hypoxia + canstatin.

It is presumed that canstatin binds α_v_β_3_ and α_v_β_5_ integrins in endothelial and tumor cells [14,15]. Therefore, we investigated whether canstatin exerts the cytoprotective effects through the interaction with α_v_β_3_ and α_v_β_5_ integrins by using cilengitide (1 μM), an α_v_β_3_ and α_v_β_5_ integrin inhibitor. Canstatin significantly inhibited the hypoxia (48 h)-induced decreases of cell viability, which was significantly suppressed by cilengitide (n = 5, p<0.01 vs. cont, p<0.01 vs. hypoxia, and p<0.01 vs. hypoxia + canstatin, Fig 2A and 2B).

### Canstatin enhances phosphorylation of FAK and Akt under hypoxia

**Fig 3:**
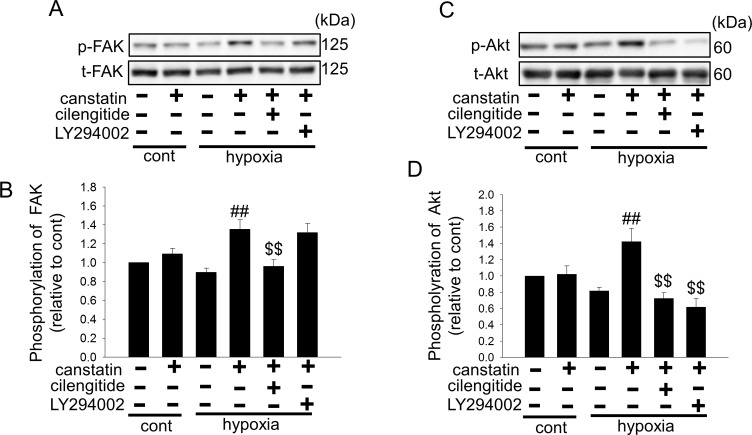
Canstatin enhances phosphorylation of focal adhesion kinase (FAK) and Akt under hypoxic condition. After the stimulation with hypoxia for 24 h in the presence or absence of canstatin (250 ng/ml), cilengitide (1 μM), or LY294002 (10 μM), an inhibitor of phosphatidylinositol-3 kinase (PI3K)/Akt pathway, total cell lysates of H9c2 cardiomyoblasts were harvested. Expression of phospho-FAK (p-FAK) and phospho-Akt (Ser473) (p-Akt) was determined by Western blotting. (A, C) Representative blots of p-FAK and total-FAK (t-FAK) (A) or p-Akt and total-Akt (t-Akt) (C) were shown. (B, D) Levels of phosphorylated proteins were corrected by total proteins, and the normalized expression relative to cont was shown as mean ± S.E.M. (B: FAK: n = 6, D: Akt: n = 4). ##: p<0.01 vs. hypoxia, $$: p<0.01 vs. hypoxia + canstatin.

α_v_β_3_ and α_v_β_5_ integrins are known to activate Akt and subsequent survival signaling pathway [25–27]. Therefore, we investigated whether canstatin affects the phosphorylation of FAK and Akt. Canstatin-alone treatment (250 ng/ml, 24 h) had no effect on the phosphorylation of FAK and Akt under normoxia (Fig 3A–3D). Interestingly, canstatin (250 ng/ml, 24 h) significantly enhanced phosphorylation of FAK (n = 6, p<0.01 vs. hypoxia) and Akt (n = 4, p<0.01 vs. hypoxia) under hypoxia (Fig 3A–3D). Cilengitide (1 μM) significantly inhibited the canstatin-induced phosphorylation of FAK (n = 6, p<0.01 vs. hypoxia + canstatin) and Akt (n = 4, p<0.01 vs. hypoxia + canstatin) (Fig 3A and 3B). We confirmed that LY29002 (10 μM), a phosphatidylinositol-3 kinase (PI3K)/Akt pathway inhibitor, significantly inhibited the canstatin-induced phosphorylation of Akt (n = 4, p<0.01 vs. hypoxia + canstatin) but not phosphorylation of FAK (Fig 3C and 3D).

### Canstatin inhibits hypoxia-induced apoptosis of H9c2 cardiomyoblasts via PI3K/Akt pathway

**Fig 4:**
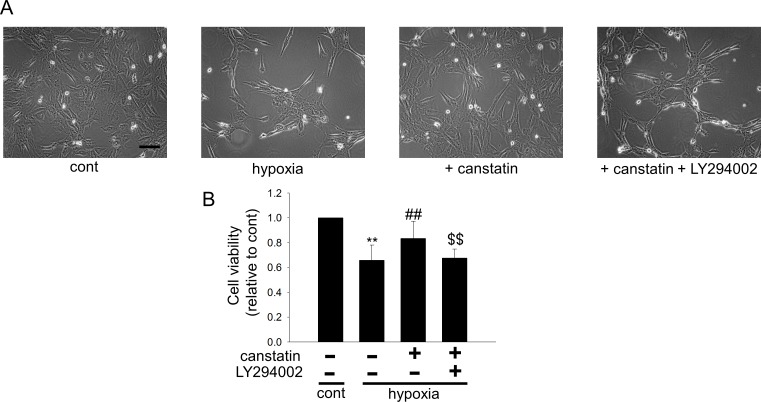
Canstatin inhibits hypoxia-induced apoptosis of H9c2 cardiomyoblasts via PI3K/Akt pathway. H9c2 cardiomyoblasts were stimulated with hypoxia for 48 h in the presence or absence of canstatin (10–250 ng/ml) and LY294002 (10 μM). (A) Representative phase-contrast microscopic images of H9c2 cells after treatment with vehicle (cont), hypoxia-alone, hypoxia + canstatin (250 ng/ml), or hypoxia + canstatin (250 ng/ml) + LY294002 (10 μM) were shown (n = 4). Scale bar represents 100 μm. (B) After the stimulation with hypoxia for 48 h in the presence or absence of canstatin and LY294002, living cell number was counted by a colorimetric method using cell counting kit-8. The normalized cell number relative to cont was shown as mean ± S.E.M (n = 6). **: p<0.01 vs. cont, ##: p<0.01 vs. hypoxia, $$: p<0.01 vs. hypoxia + canstatin.

We next investigated whether canstatin exerts the cytoprotective effects via PI3K/Akt pathway by using LY294002 (10 μM). Canstatin significantly inhibited the hypoxia (48 h)-induced decreases of cell viability, which was significantly suppressed by LY294002 (n = 6, p<0.01 vs. hypoxia + canstatin, Fig 4A and 4B).

### Recruitment of α_v_ integrin to the focal adhesion

**Fig 5:**
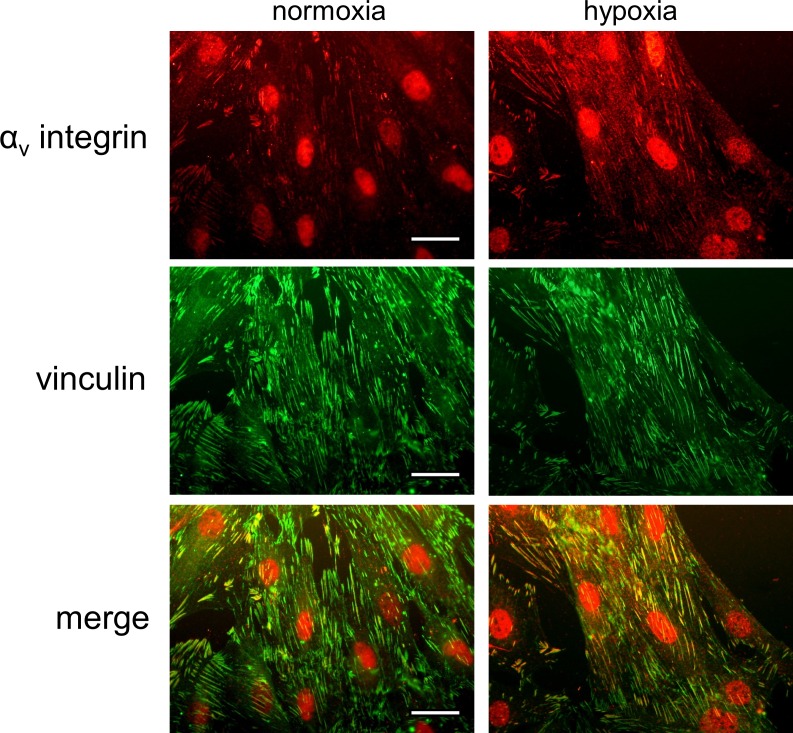
Hypoxia induces translocation of αv integrin to focal adhesion. H9c2 cardiomyoblasts were stimulated with normoxia or hypoxia for 24 h. Then a double immunocytochemical staining of αv integrin and vinculin was performed. Representative images of immunofluorescence staining of αv integrin (red), vinculin (green) and merge in H9c2 cells under normoxia (left) or hypoxia (right) were shown (n = 4). A yellow color represents co-localization of αv integrin and vinculin. Scale bar represents 50 μm.

It has been reported that hypoxia induces the recruitment of α_v_β_3_ and α_v_β_5_ integrins to cell membrane in tumor cells [22,23]. We finally investigated whether hypoxia stimulation causes a localization of α_v_ integrin to focal adhesion in H9c2 cardiomyoblasts by performing a double immunofluorescence staining of α_v_ integrin and vinculin, a focal adhesion marker. Co-localization of α_v_ integrin and vinculin was observed prominently in hypoxia (24 h)-stimulated H9c2 cardiomyoblasts compared with normoxia (n = 4, Fig 5). Canstatin and/or cilengitide did not affect the hypoxia-induced recruitment of α_v_ integrin to focal adhesion (data not shown, n = 4).

## Discussion

**Fig 6:**
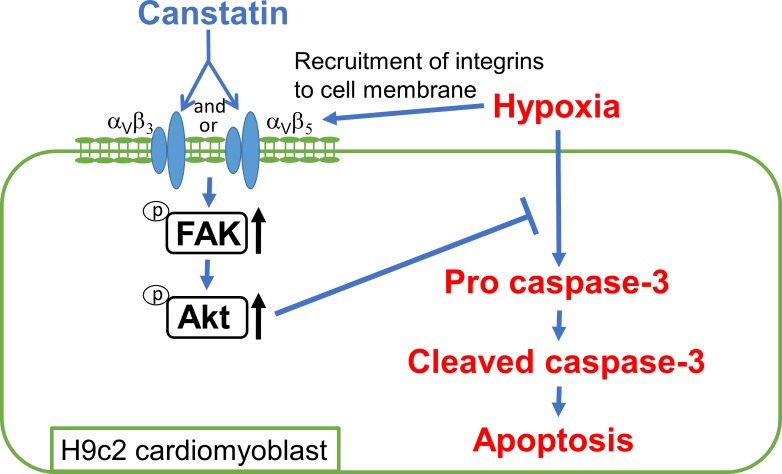
Schematic model for cytoprotective effects of canstatin on hypoxia-induced apoptosis of H9c2 cardiomyoblasts. Hypoxia induces the recruitment of integrins to cell membrane and causes apoptosis through the activating caspase-3 cascade. Canstatin inhibits the hypoxia-induced apoptosis through phosphorylation of FAK and Akt by activating α_v_β_3_ and/or α_v_β_5_ integrins in H9c2 cardiomyoblasts.

In this study, we for the first time demonstrated that canstatin has a cytoprotective effect on hypoxia-induced apoptosis in H9c2 cardiomyoblasts through the activation of α_v_β_3_ and/or α_v_β_5_ integrins and the phosphorylation of FAK and Akt. (Fig 6).

Canstatin is produced by cleaving type IV collagen α2 chain which is ubiquitously expressed around cardiomyocytes. To the best of our knowledge, the blood concentration of canstatin in a living body has not been clarified. Hamano et al. reported that the blood concentration of tumstatin, an α3 chain cleaved product of type IV collagen, is 336±28 ng/ml in a normal mouse [32]. Thus, we thought that the physiological level of canstatin is ng/ml order and used the concentrations of canstatin ranging from 10 to 250 ng/ml in this study.

Firstly, we confirmed that canstatin-alone treatment had no effect on the morphology of H9c2 cardiomyoblasts. As with the previous report [33], hypoxia significantly increased cleaved caspase-3 expression and caused apoptosis in H9c2 cardiomyoblasts. In this study, canstatin suppressed the hypoxia-induced several apoptotic features, such as morphological damages, decreases of cell viability and increased cleaved caspase-3 expression, in a concentration-dependent manner. We previously reported that canstatin inhibited the isoproterenol-induced apoptosis in differentiated H9c2 cells [29], supporting the present findings that canstatin has a cytoprotective effect in cardiac cells. While canstatin is known to exert a pro-apoptotic effect on endothelial and tumor cells [11–16], it showed the opposite effects on the H9c2 cardiomyoblasts. The reason is speculated that the concentration of canstatin used in this study is much lower than the concentration of canstatin (15–20 μg/ml) reported to stimulate apoptosis [11–13,16]. Kamphaus et al. reported that 1 μg/ml canstatin can inhibit proliferation of human umbilical vein endothelial cells (HUVECs), while it (up to 40 μg/ml) had no significant effect on the proliferation of renal carcinoma cells (786–0), prostate cancer cells (PC-3) or human embryonic kidney cells (HEK 293) [11]. We found that canstatin (1 μg/ml) under normoxia had no effect on cell viability (n = 6). It is thus suggested that lower concentration of canstatin might exert cytoprotective effects through the different mechanisms from endothelial cells.

The receptor for canstatin in endothelial and tumor cells was proposed as α_v_β_3_ and α_v_β_5_ integrins [14,15] and these integrin subtypes are expressed in cardiomyocytes [19–21]. Therefore, we next investigated whether canstatin exerted the cytoprotective effects against hypoxia through integrins and integrin-related signal pathway in H9c2 cardiomyoblasts. Cilengitide, an α_v_β_3_ and α_v_β_5_ integrin inhibitor, significantly suppressed the cytoprotective effect of canstatin. It has also been reported that FAK/Akt pathway, which is involved in cell survival, exists downstream of α_v_β_3_/α_v_β_5_ integrins [25–27]. Therefore, we further investigated whether canstatin activates FAK/Akt signaling. While canstatin-alone treatment had no effect on the phosphorylation of FAK and Akt under a normoxic condition, canstatin significantly enhanced phosphorylation of FAK and Akt under a hypoxic condition. In the present study, the mRNA expression of α_v_, β_3_ and β_5_ integrins was not elevated by hypoxia for 24 h (data not shown, n = 3). It has been reported that hypoxia induced the recruitment of α_v_β_3_ and α_v_β_5_ integrins to cell membrane in tumor cells [22,23]. In this study, we confirmed that hypoxia enhanced the recruitment of α_v_ integrin to focal adhesion which was determined by a co-immunostaining with vinculin, a focal adhesion marker protein. Previous study has shown that the overexpression of α_v_β_5_ integrin induced the phosphorylation of FAK in human dermal myofibroblasts [34]. The activation of integrin/FAK/Akt pathway was also shown to be regulated by conformational change of integrin as well as the quantity and clustering of integrin [35]. The conformational change of integrin has been reported to be induced by inside-out signaling derived from various exogenous stimulations. Hypoxia has also been reported to convert the integrin conformation to active form in myocardial cells [36]. Thus, it is proposed that the recruitment to the focal adhesion and conformational change of integrins may be responsible for the activation of FAK/Akt signaling by canstatin under the hypoxic condition but not under the normoxic condition in H9c2 cardiomyoblasts.

We also showed that cilengitide significantly suppressed the phosphorylation of FAK and Akt. Further, LY294002, an inhibitor of PI3K/Akt pathway, significantly suppressed the cytoprotective effect of canstatin on the hypoxia-induced apoptosis. It has been reported that Akt inactivates the caspase pathway by phosphorylating pro-apoptotic proteins like Bad and inhibiting the release of cytochrome c from mitochondria [37,38]. Collectively, canstatin might inhibit the hypoxia-induced caspase-dependent apoptosis pathway through the activation of FAK/Akt signaling via activating α_v_β_3_ and α_v_β_5_ integrins in H9c2 cardiomyoblasts. The integrin ligands containing Arg-Gly-Asp (RGD) are shown to bind a binding pocket, an extracellular domain of integrin α and β chains [39,40]. Although cilengitide has the RGD motif, recombinant canstatin used in this study does not have it. Kireeva et al. reported that CCN1, a matricellular protein, which does not have RGD sequence, adhered to HUVECs via α_v_β_3_ integrin and the adhesion was inhibited by Arg-Gly-Asp-Ser (RGDS) peptides [41]. They also indicated that the binding of RGDS on α_v_β_3_ integrin exerted conformational changes which caused a masking of other binding sites on the receptor for non-RGD-containing ligands [41]. Thus, cilengitide may change the conformation of α_v_β_3_ and/or α_v_β_5_ integrins by binding and inhibit the activation of canstatin.

Canstatin has been reported to inhibit FAK/PI3K/Akt signaling in HUVECs [16]. In the present study, however, canstatin activated FAK/Akt signaling in H9c2 cardiomyoblasts. Legler et al. reported that a cyclic RGD-peptide, which binds α_v_β_3_ integrin, has biphasic effects (an antagonistic phase at high concentrations and an agonistic phase at low concentrations) [42]. It has also been reported that higher concentration (≥ 20 μM) of cilengitide has an anti-angiogenic, while lower concentration (0.2–20 nM) of cilengitide enhances the growth of tumors by promoting vascular endothelial growth factor-mediated angiogenesis [43]. It is thus suggested that lower concentration of canstatin used in the present study might exert agonistic effects on α_v_β_3_ and α_v_β_5_ integrins. Further studies regarding the effects of higher concentration of canstatin on H9c2 cardiomyoblasts might help to clarify the discrepancy.

In conclusion, we for the first time revealed that canstatin inhibits hypoxia-induced apoptosis via FAK and Akt pathways through activating α_v_β_3_ and/or α_v_β_5_ integrins in H9c2 cardiomyoblasts (Fig 6). Our findings indicate canstatin as a new drug target in ischemic heart disease due to its cardioprotective effects against hypoxic stress-induced apoptosis.
